# A modular approach to map out the conformational landscapes of unbound intrinsically disordered proteins

**DOI:** 10.1073/pnas.2113572119

**Published:** 2022-06-03

**Authors:** Thinh D. N. Luong, Suhani Nagpal, Mourad Sadqi, Victor Muñoz

**Affiliations:** ^a^Center for Cellular and Biomolecular Machines, University of California, Merced, CA 95343;; ^b^Chemistry and Biochemistry Graduate Program, University of California, Merced, CA 95343;; ^c^Bioengineering Graduate Program, University of California, Merced, CA 95343;; ^d^Department of Bioengineering, University of California, Merced, CA 95343

**Keywords:** intrinsically disordered proteins, folding upon binding, folding landscapes, conformational rheostat

## Abstract

Intrinsically disordered proteins (IDPs) fold upon binding to select/recruit multiple partners, morph around the partner's structure, and exhibit allostery. However, we do not know whether these properties emerge passively from disorder, or rather are encoded into the IDP's folding mechanisms. A main reason for this gap is the lack of suitable methods to dissect the energetics of IDP conformational landscapes without partners. Here we introduce such an approach that we term molecular LEGO, and apply it to NCBD, a helical, molten globule–like IDP, as proof of concept. The approach entails the experimental and computational characterization of the protein, its separate secondary structure elements (LEGO building blocks), and their supersecondary combinations. Comparative analysis uncovers specific, yet inconspicuous, energetic biases in the conformational/folding landscape of NCBD, including 1) strong local signals that define the three native helices, 2) stabilization of helix–helix interfaces via soft pairwise tertiary interactions, 3) cooperative stabilization of a heterogeneous three-helix bundle fold, and 4) a dynamic exchange between sets of tertiary interactions (native and nonnative) that recapitulate the different structures NCBD adopts in complex with various partners. Crucially, a tug of war between sets of interactions makes NCBD gradually shift between structural subensembles as a conformational rheostat. Such conformational rheostatic behavior provides a built-in mechanism to modulate binding and switch/recruit partners that is likely at the core of NCBD's function as transcriptional coactivator. Hence, the molecular LEGO approach emerges as a powerful tool to dissect the conformational landscapes of unbound IDPs and rationalize their functional mechanisms.

## Significance

Intrinsically disordered proteins have the unique ability to morph in response to multiple partners and thereby process sophisticated inputs and outputs. It is, however, a mystery whether their response is passive, that is, entirely determined by the partner, or controlled via an internal, yet unknown, folding mechanism. Here we introduce an approach to examine this key question and demonstrate its potential by dissecting the conformational properties of the partially disordered protein NCBD and obtaining important clues about how it performs its biological function.

The traditional biochemical paradigm states that protein sequences are encoded to fold into thermodynamically stable three-dimensional (3D) structures that define their biologically functional states (1). However ∼40% of the human proteome appears to be composed of protein domains/regions that are intrinsically disordered (IDPs or IDRs) (2, 3). IDPs are paradigm challengers because they are disordered in their resting state (4, 5), fold, completely or partially, upon binding to their biological effectors (6, 7), can bind structurally diverse partners (8, 9), and exhibit allostery without quaternary or even defined tertiary structure (10, 11). IDPs are more abundant in higher-order organisms, in whom they play key regulatory roles for essential biological processes (12). From a physical viewpoint, IDPs have distinct sequence patterns (13), including high net charge, low hydrophobicity, and enriched proline content (2, 14). Some IDPs are devoid of any structure, even after binding to partners (15), but many are partially disordered (IPDP) and morph to accommodate their partners. Hence, efforts have focused on investigating their folding upon binding (6, 10, 16–18). These studies have shown that IPDPs bind partners via conformational selection (fold first and then bind) or induced-fit (bind first and fold while bound) processes. However, what remains a mystery is the role (if any) that the folding mechanism of the IPDP plays in defining its binding/functional properties. For instance, structural disorder is often considered sufficient to enable the IPDP to morph into any required shape on cue. But, if so, how does an IPDP manage to bind specifically, select among partners, and exhibit allostery? In addition, folding upon binding is often interpreted as a binary transition (conformational switch). Such transitions require simultaneous folding and binding (19), which contradicts findings of IPDPs binding via induced fit (20, 21) or alternating between conformational selection and induced fit (7, 22). Moreover, to fold upon binding as a conformational switch, IPDPs sequences would need to fully encode all the structures they form in complex with diverse partners.

A possible solution to these puzzles is for IPDPs to fold upon binding as conformational rheostats (CR) (23), a functional mechanism linked to the gradual structural transitions of downhill folding (24). Downhill domains have IDP-like sequences and are mostly stabilized by local interactions, which makes them fold fast but also marginally unstable, and hence partially disordered (23). The key to CR function is a flexible conformational ensemble with built-in energetic biases toward specific (potentially multiple) subensembles. Such biases would provide the driving force for selecting partners and allostery, whereas the gradual conformational transitions can explain how IPDPs morph around diverse partners and combine conformational selection and induced-fit binding (23). The connections between downhill folding and IPDP binding have been explored using computational approaches (19, 25, 26). However, to establish whether the folding mechanism is what controls IPDPs’ binding and function, it is essential to resolve the conformational landscapes and energetics of the IPDP in the absence of partners. Achieving this by experiment has been a major hurdle. The standard approach to investigate protein conformational ensembles relies on thermodynamic and/or kinetic measurements of the (un)folding transition and their analysis with a two-state model (unfolded and native) to determine the changes in free energy upon folding and unfolding, and in equilibrium (27). When performed on collections of select mutants, these experiments provide local perturbation maps that can be used to infer the folding landscape (28). The analysis requires a cooperative (un)folding transition with well-defined ends from which to determine and extrapolate the properties of the interconverting states. For IDPs, this key requirement is met when folding is induced by binding using the partner’s concentration as a thermodynamic variable (16, 17), but not in the absence of a partner. Even partially structured IPDPs exhibit transitions that are too broad and uncooperative for such an approach (29). As a consequence, the folding landscapes of IDPs without partners have only been accessible via molecular simulations (26, 30–32). Such simulations have led to important insights, but it is essential to cross-check them by experiment at levels comparable to what has been recently attempted for IDP folding upon binding (33).

In response to this challenge, we introduce here a modular approach that we term molecular LEGO. The approach starts by decomposing an IPDP into its basic secondary structural elements, or LEGO building blocks, and their combinations. The combined elements recapitulate subsets of tertiary interactions, in analogy to the complementary indentations between bricks in the LEGO toy. The molecular LEGO is inspired by work in the early 1990s that searched for local folding nuclei on two-state folding proteins (34), and which revealed weak local biases (34) and the need for nearly the entire protein to elicit detectable folding (35). A more recent study on the IDP ACTR has shown similarly weak local conformational biases (36). The dissection of an IDP into structural elements has also been used in molecular simulation studies to facilitate conformational sampling via the much faster dynamics of the fragments (37). The key addition here is the comparative quantitative analysis of hierarchically organized protein segments via experiments and simulations. In this regard, the conformational analysis of the building blocks probes local interactions, but also provides reference ensembles for interpreting the properties of higher-order fragments. Such reference ensembles are essential to reliably detect the subtle biases expected on IPDPs, and to convert them into energetic contributions using simple statistical thermodynamic analysis. We contend that such a modular approach can provide new key insights about the tertiary interactions and cooperative energetics that stabilize IPDP folding ensembles in the absence of partners. To demonstrate this assertion, we focused on the protein NCBD. NCBD is categorized as IPDP, and there is a wealth of biophysical data available on its folding and binding to compare with, including nuclear magnetic resonance (NMR) (29, 38), molecular simulations (25, 31), and single-molecule fluorescence resonance energy transfer (39–41). NCBD binds to multiple, structurally diverse partners, including IDPs [e.g., p53-TAD (38) and ACTR (8)] and globular proteins such as IRF3 (42), by adapting its ensemble to the partner's properties. In its free form, NCBD exhibits high α-helical content without defined tertiary structure, but it forms a dynamic three-helix bundle driven by a few midrange contacts (29). Critically, the (dis)ordering transitions of NCBD are broad and featureless, including its thermal unfolding and stabilization via the cosolvent trifluoroethanol (TFE) (*SI Appendix*, Fig. S1). All these properties make NCBD ideal for a molecular LEGO proof of concept.

## Results

### Molecular LEGO Design.

**Fig. 1.**
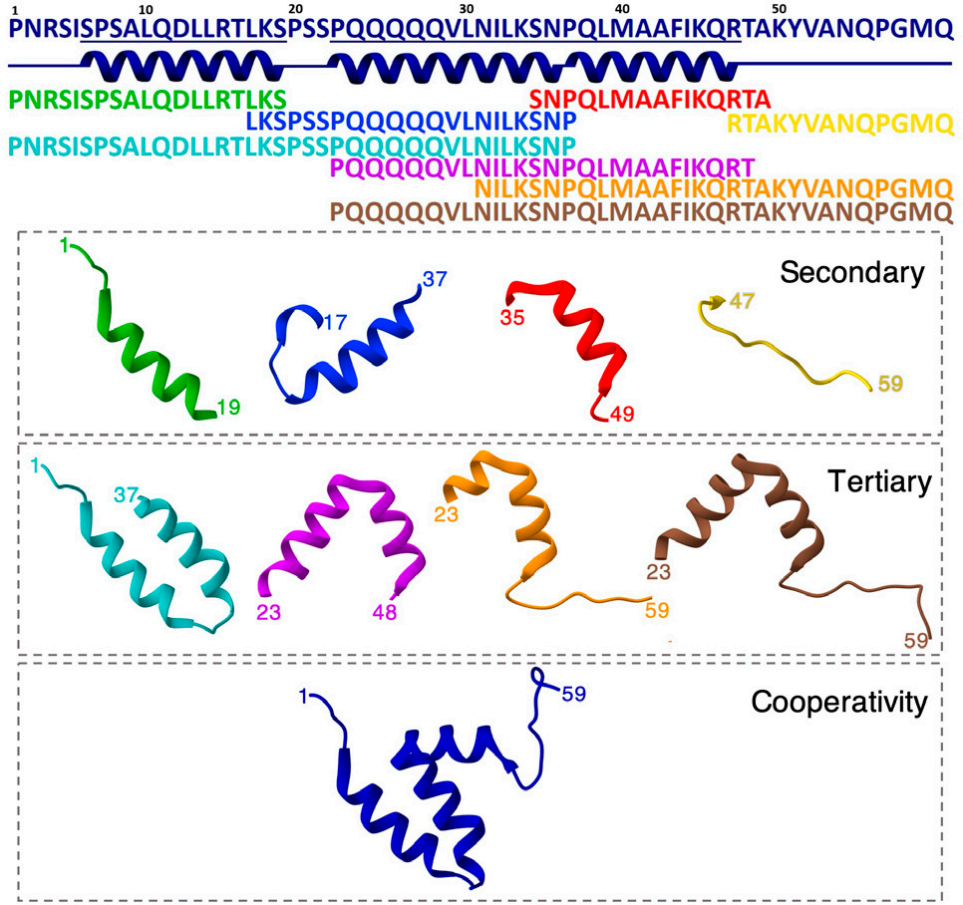
Molecular LEGO design. (*Top* to *Bottom*) The complete NCBD sequence (ID: 2KKJ) and a diagram showing the three α-helices of the NMR ensemble in navy blue. Sequences of the eight LEGO elements: building blocks in primary colors (H1, green; H2, blue; H3, red; T, yellow), and combined elements in secondary colors (H1–H2, cyan; H2–H3, magenta; H3–T, orange; H2–H3–T, brown). (*Bottom*) Sketch showing the structure of each fragment and full NCBD (same color code). The building blocks report on secondary structure propensities, and their combinations on pairwise tertiary interactions, e.g., H1–H2 reports on the interactions between helices 1 and 2. Comparison with the full protein reports on the degree of cooperativity.

The design of the LEGO elements (locations and extension along the sequence) on highly disordered proteins is far from trivial, unless there are available structures in complex with partners. IPDPs, however, do have residual structure, which, for NCBD, was sufficient to enable the determination on an NMR ensemble based on chemical shifts and a few midrange Nuclear Overhauser Effects (NOE) (29). We used this NMR ensemble to divide the 59-residue sequence of NCBD into four building blocks that represent its local (secondary) structural segments: helices 1, 2, and 3 (H1, H2, and H3) and the C-terminal tail (T). We further refined the limits of the α-helical regions based on predictions of helical propensity from the prediction algorithm AGADIR (43), which delineate a distinct helix profile (*SI Appendix*, Fig. S2). We then designed four combinations of consecutive building blocks (H1H2, H2H3, H3T, and H2H3T) that recapitulate the various sets of “native” pairwise tertiary interactions. Finally, the comparison of LEGO elements with the entire protein is expected to inform on the overall contribution from global cooperativity. The complete molecular LEGO design of NCBD is shown in Fig. 1.

### Analysis of Conformational Ensembles.

**Fig. 2.**
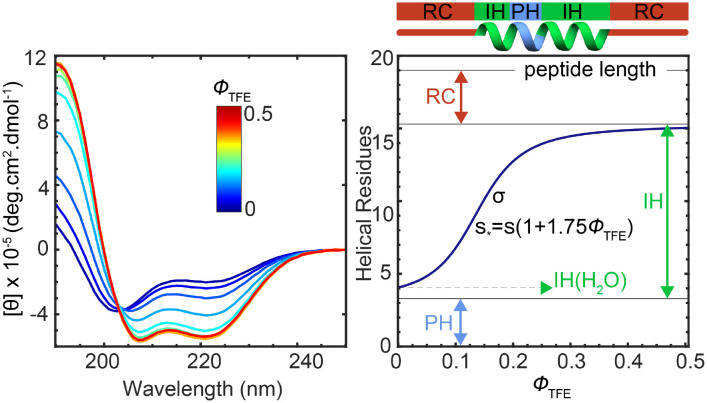
Experimental conformational analysis. (*Left*) CD spectra of H1 as a function of TFE volume fraction (ϕ_TFE_). (*Right*) Tripartite helix–coil analysis. At the top is an exemplary peptide with PH, IH, and RC units. TFE increases elongation (*s*) of IH units in sequence independent fashion. The average number of helical residues obtained from CD (dark blue) is fit to *SI Appendix*, Eq. **S7** to obtain σ, *s*, IH, and PH. RC is obtained as RC=N−IH−PH.

We analyzed NCBD and its LEGO elements by experiment and simulation. Experimentally, we employed far-ultraviolet (far-UV) circular dichroism (CD) spectroscopy, which reports on the average peptide bond conformation and is particularly sensitive to α-helical structure (NCBD and most IPDPs are, or become upon binding, α-helical). We use the cosolvent 2,2,2-TFE as a structure-promoting agent. TFE is a polar/organic cosolvent that induces local structure in peptides and proteins by strengthening the backbone intramolecular hydrogen bonds (44). TFE has been widely used as a helix-promoting agent (45), but is also known to stabilize β-hairpin structures (46, 47) and to promote hydrophobic interactions by changing the hydration shell (48). The TFE CD titration of H1 is given in Fig. 2, *Left* as an example. In the absence of TFE, the CD spectrum of H1 indicates ∼20% α-helix, with the remainder being random coil (RC). TFE addition steadily increases the α-helical content of H1 until it plateaus (beyond 0.3 ϕ_TFE_). Although quantitatively different, the TFE titrations of all the other LEGO elements and full NCBD share the same features (all data are shown in *SI Appendix*, Fig. S3). These results indicate that all these TFE titrations can be analyzed in terms of the helix–coil transition, which describes α-helix formation as the interplay between nucleation (*σ*) and elongation (*s*) (49). The effect of TFE on helix formation can be simply described as an enhancement in elongation (larger *s*) due to stronger hydrogen bonds, and hence as sequence independent. Here we used $s\left(\text{TFE}\right)=2.75s\left({\text{H}}_{2}\text{O}\right)$, or a ∼1 RT stabilization, for all the molecules. The effective $s_{*}$ at each TFE volume fraction can be calculated as the weighted average of both *s* values according to the composition of the mixed solvent (1 − ϕ_TFE_ and ϕ_TFE_) as shown in the Fig. 2, *Right* equation (*SI Appendix*). When the polypeptide has sufficiently high *σ* and *s* parameters in water, the addition of TFE promotes a cooperative (sigmoidal) transition to α-helical structure (Fig. 2, *Right*). In this case, however, it is not appropriate to use a homopolymer helix/coil model, because the NCBD sequence is highly heterogeneous (Fig. 1). To describe how such heterogeneity can affect the average helical content as a function of TFE (CD only reports the average peptide bond conformation), we implemented a tripartite helix–coil model based on the original Zimm–Bragg treatment (50). The tripartite model discretizes the helical propensity spectrum of a heteropolypeptide chain into three types of units (peptide bonds): preformed helix (PH), which are already α-helical without TFE; RC, which are random coil regardless of TFE; and inducible helix (IH), which have a residual α-helix population that is enhanced by TFE (Fig. 2, *Right*). The model defines the average number of helical peptide bonds on any peptide/protein with four parameters: the number of PH units, number of IH units and σ, s for the IH units (Fig. 2, *Right*); that is only one more parameter than a standard homopolymer helix–coil model. The tripartite model fits the data of all the NCBD molecules much better than the three-parameter homopolymer model, with an improved performance that is statistically significant at >99% confidence according to the *F* test (*SI Appendix*).

**Fig. 3.**
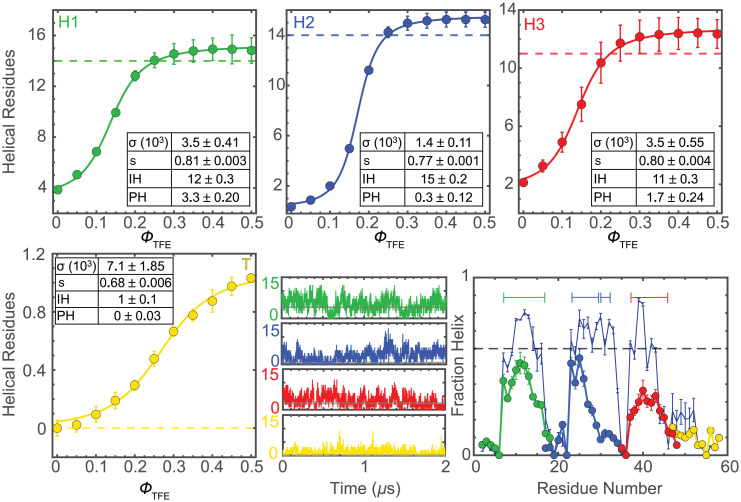
LEGO building blocks. Colors as in Fig. 1. Experimental number of helical residues of (*Top Left*) H1, (*Top Middle*) H2, (*Top Right*) H3, and (*Bottom Left*) T as a function of ϕ_TFE_. Error bars indicate 1 SD from two independent experiments. The curves represent fits to *SI Appendix*, Eq. **S7**; fitted parameters and fitting errors (1 SD) are given in *Insets*. Dashed lines indicate the helix length in the NMR structure. (*Bottom Middle*) Number of helical residues as a function of time for one exemplary MD trajectory (all data are provided in *SI Appendix*, Fig. S5). The horizontal gray line indicates the experimental value at Φ_TFE_ = 0. (*Bottom Right*) Helix fraction per residue from MD simulations. NCBD's profile is shown with a thin navy-blue line for reference. Horizontal bars signal the average helix length (consecutive residues with > 0.1 helix). The gray dashed line signals 60%.

**Fig. 4.**
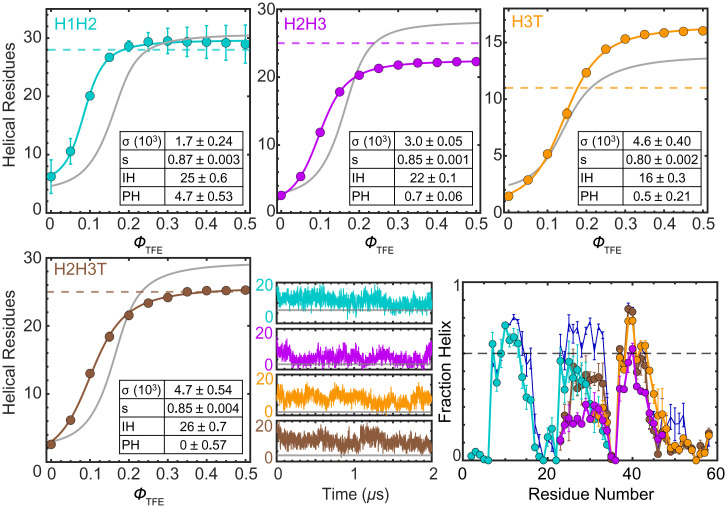
LEGO combinations. Colors as in Fig. 1. Experimental number of helical residues of (*Top Left*) H1H2, (*Top Middle*) H2H3, (*Top Right*) H3T, and (*Bottom Left*) H2H3T as a function of ϕ_TFE_. Error bars, curve fits, parameters, fitting errors, and dashed lines are as in Fig. 3. The gray curves show the compounded curves of the relevant building blocks (e.g., H1 and H2 for H1H2). (*Bottom Middle*) Number of helical residues as a function of time for one exemplary MD trajectory (all data are provided in *SI Appendix*, Fig. S5). (*Bottom Right*) Helix fraction per residue from MD simulations. Error bars, symbols, and lines are as in Fig. 3.

**Fig. 5.**
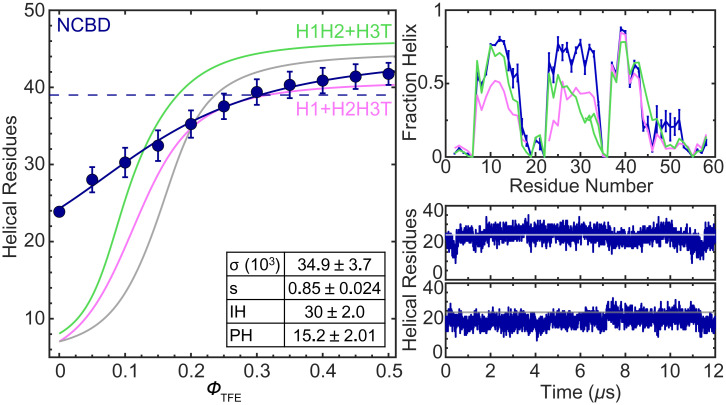
Full NCBD ensemble. (*Left*) Experimental number of helical residues of full NCBD as a function of ϕ_TFE_. Error bars, curve fits, parameters, fitting errors, and dashed line are as in Fig. 3. The gray curve shows the compounded H1, H2, H3, and T curves. Pink is H12 plus H3T, and green is H1 plus H23T. (*Right*) Helix fraction per residue (*Top*) and number of helical residues (*Bottom*) as a function of time from two MD trajectories. Error bars, symbols, and lines are as in Fig. 3; pink and green are as in *Left*.

We also performed atomistic MD simulations in explicit solvent: two independent 12-μs trajectories for NCBD and two or three sets of 2-μs trajectories for each LEGO element, as we expected faster conformational dynamics on them. We used the CHARMM22* force field with TIP3P water, which has been found suitable for partially disordered proteins (51, 52). We first examined the MD simulations using the fraction of native contacts (*Q*) as an order parameter (*SI Appendix*, Fig. S4). The LEGO building blocks showed sharp fluctuations in *Q* (they have few native contacts) that take place in tens of nanoseconds. The combined LEGO elements exhibited *Q* fluctuations of smaller amplitude and slower dynamics, but several transitions were still observable in each 2-μs trajectory (*SI Appendix*, Fig. S4). The behavior of NCBD is similar, although with an additional slowdown: Six-times-longer trajectories produce similar numbers of transitions. The observation of several transitions per trajectory and the consistency between independent trajectories suggest that conformational sampling within these timescales is reasonable. We then computed the fraction helix, and nucleation and elongation parameters, for each peptide bond in each molecule. The agreement between the residue-specific helix populations obtained from independent simulations (Figs. 3–5 and *SI Appendix*, Fig. S5) further supports that the simulated timescales afford reasonable sampling. The fraction helix profiles of the LEGO elements and NCBD are given in Figs. 3–5 and *SI Appendix*, Fig. S6.

### Conformational Propensities of LEGO Building Blocks.

In general, we find that the three regions containing α-helices in the native NMR ensemble have residual helical structure and are highly sensitive to TFE (Fig. 3). H1 has the highest residual helical structure, in both experiments and simulations. The maximal helix lengths (i.e., at the highest ϕ_TFE_) are just one residue longer than in the NMR ensemble, which indicates that the three NCBD helices are defined by strong local signals. The tail (T) does not have a detectable helix, but forms a single helical turn (i.e., one hydrogen-bonded unit) at the highest ϕ_TFE_. The TFE transitions are well reproduced by the tripartite helix–coil model, which reveals that the costs of nucleation (*σ*) are close to the values for polyalanine-based peptides (53). H1 and H3 are slightly easier to nucleate and hence less cooperative than H2. Elongation is slightly <1 for all the peptides, which explains their residual helix (on an infinitely long helix, s=1 results in 50% helix), but also their high sensitivity to TFE. T is disordered but contains a short region that is primed to become helical by stabilizing factors.

The MD simulations are in good agreement with the experimental findings, including the average helix content per molecule (particularly H1 and H3), and the presence of marginal helical propensity in T. They also show nonuniform helix populations, hence further supporting the analysis of the experiments with the tripartite helix–coil model. The helical regions in simulations are also in excellent agreement with the NCBD NMR ensemble, confirming the presence of strong local signals. In contrast, the simulations produce systematically lower nucleation costs (about fivefold to 10-fold larger *σ*) and less propensity to elongate (smaller *s*). Interestingly, the differences in *σ* and *s* compensate each other to produce similar helical contents (Fig. 3). The implication is that the combination of force field and water model used here underestimate the cooperativity of the helix–coil transition, and, generally, of folding, a result that is consistent with previous comparative studies (54).

### Estimating Pairwise Tertiary Interactions.

The results of the combined LEGO elements are qualitatively similar: 1) residual helical structure in native conditions, 2) strong response to TFE, 3) sigmoidal TFE transitions, and 4) agreement with the helix lengths in the NMR ensemble (Fig. 4). However, the comparison between combined LEGO elements and the compounded effects of their individual building blocks (gray curves) reveals significant contributions from tertiary interactions. For instance, the combined elements exhibit enhanced sensitivity to TFE, as manifested by sharper slopes and reaching a plateau at lower ϕ_TFE_, and hence larger *σ* and *s*, albeit the experiments do not detect marked net increases of helical structure in water. This indicates that each set of pairwise tertiary interactions is insufficient to significantly increase the helix population on its own. The simulations do show enhanced helical content, possibly owing to their much higher sensitivity and resolution. Another observation is that the thermodynamic coupling between consecutive LEGO building blocks has significant impact on redefining the maximal helix lengths, most notably of H3.

On an individual basis, we find that the interactions between helices 1 and 2 are stronger than between 2 and 3. H1H2 does, in fact, exhibit enhanced helical content also in experiments, in excellent agreement with the simulations (cyan in Fig. 4). The effects on H2H3 are more subdued in simulations and only detectable from the TFE response in experiments. The impact of the tail on helix 3 is interesting, as the extended C-terminal sequence stimulates the growth of the helix beyond that found in the NMR ensemble. Helix extension is clear in experiments (three more residues) and simulations (see H3T in orange in Fig. 4). In other words, the tail does not nucleate helix structure on its own, but it extends a helix coming from the preceding sequence. The simulations indicate that this effect is purely driven by local interactions (helix–coil cooperativity). The extension of H3 onto the tail is also predicted by AGADIR (*SI Appendix*, Fig. S2), further supporting its local origin.

Pairwise interactions do have distinct effects on defining the length of the helices. For instance, the interactions between helices 1 and 2 do not change the length of either helix in experiments or simulations. In contrast, experiments on H2H3 indicate a maximal helix of ∼23 residues (vs. 25 in the NMR ensemble) and ∼28 in the sum of H2 and H3. This difference seems to arise, in part, from helix capping effects of the region connecting helices 1 and 2, which is absent in H2H3 and H2H3T (Fig. 1). This effect is also evident in the simulations, which show some helix population in that connecting region, as well as the stabilization of the beginning of helix 2 in H2 relative to H2H3 (Fig. 3 vs. Fig. 4). The experiments also show that helix 2 impedes the elongation of helix 3 into the tail: H2H3T has a maximum helix of 26, in perfect agreement with the NMR ensemble, whereas H2 and H3T add up to ∼30. The same pattern is observed in simulations, which show a longer third helix in H3T than in H2H3T. Strikingly, the simulations also reveal *“*nonnative*”* effects of the tail, which stabilizes helices 2 and 3 without becoming itself helical (brown vs. orange in Fig. 4). Experiments confirm this observation, showing enhanced elongation (*s*) and reduced helix length of H2H3T vs. H2 + H3T. The main discrepancy between experiments and simulations is quantitative: The helix stabilization induced by the tail is stronger in simulations. Hence, the simulations overestimate the helical content, most particularly for H3T and H2H3T, and, to a lesser extent, H2H3.

### Global Stabilization of the NCBD Ensemble.

The LEGO results provide a reference to interpret the uncooperative (nonsigmoidal) TFE transition of full NCBD, which is, in fact, much broader than those of its elements (Fig. 5). Compounding different LEGO elements, we can establish the behavior expected from only local interactions (gray), or after adding the interactions between helices 1 and 2 (green), or between helices 2 and 3 and tail (pink). This comparison demonstrates that NCBD has much higher helical content than the sum of its parts: ∼24 helical residues in water relative to 6 to 7 residues for the three combinations (Fig. 5). Helix–coil analysis indicates that ∼15 residues are fully helical (PH) in water, whereas the remainder comes from the partial helical population (∼30%) of many additional IH residues. Hence, in NCBD, the helix-inducible residues (IH) already have high helical content in water, which enormously facilitates nucleation: 10-fold higher *σ* relative to the LEGO elements. Elongation (*s*) is, on the other hand, minimally higher. In other words, the low TFE sensitivity of NCBD is not because its conformational ensemble is disordered, but because it is already highly primed toward forming α-helical structure via interactions that can only be formed in the entire protein. The effect of TFE on folded globular proteins is complex: It switches from native stabilizing at low volume fractions to denaturing as TFE becomes the main solvent (44). In NCBD, we see that the native-stabilizing effect extends farther in TFE concentration. Indeed, at 0.5 ϕ_TFE,_ NCBD reaches ∼41 helical residues, in agreement with the NMR ensemble (dashed line in Fig. 5). However, the helix–coil parameters indicate that helix content keeps growing beyond this point (∼four more residues), hence starting to promote nonnative conformations. Such an extended native-stabilizing range for TFE could reflect the fact that NCBD is inherently α-helical and lacks a defined hydrophobic core (44). This property could be common to other IPDPs.

For NCBD, the simulations closely reproduce the main experimental results: helical content in water (Fig. 5), nucleation, and elongation (*SI Appendix*, Table S2). The simulations also show that helix 2, which has the lowest intrinsic propensity of the three (Fig. 3), is preferentially stabilized in the full protein (Fig. 5), and engages in frequent interactions with the other two helices. The stabilization of helix 2 in the presence of both flanking helices is evident in the NCBD helix profile relative to the H1H2+H3T (green) and H1+H2H3T (pink) compounded profiles. This comparison also highlights that helix 1 is mostly stabilized by interactions with helix 2, and helix 3 is stabilized/delimited by its interplay with helix 2 and tail. The NCBD simulations also show the transient formation of many long-range interactions that were not detected in the NMR ensemble (“nonnative”), particularly between the tail and helix 1, and between helices 1 and 3. These interactions are not native but are still consistent with an antiparallel helix bundle fold. Moreover, they contribute to stabilize the helical structure of the NCBD ensemble. For instance, interactions with helix 1 make the tail regain helix structure that is suppressed by helix 2 (Fig. 5). Transient interactions between helices 1 and 3, which were not found by NMR (29), also contribute to stabilize the three-helix bundled ensemble in the simulations.

### Interaction Network and Cooperativity.

**Fig. 6.**
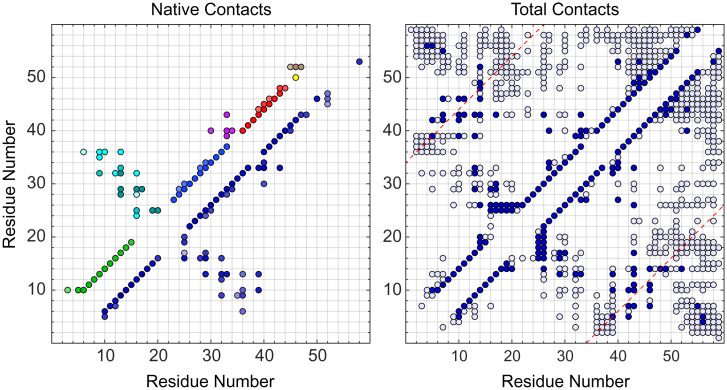
Residue–residue interaction maps. Time-averaged residue–residue contacts in the NCBD ensembles. (*Left*) Native contacts (found by NMR). Top left triangular area shows the contacts on the combined LEGO elements (local contacts in the color of the building block), and bottom right shows the contacts on full NCBD. Color intensity reflects contact probability in logarithmic scale: lightest shade for 10^−4^ ≥ *p* < 10^−3^ to darkest for 10^−1^ ≥ *p* < 1. (*Right*) Total contacts observed in full NCBD parsed in two levels: dark for 10^−1^ ≥ *p* < 1 and light for 10^−2^ ≥ *p* < 10^−1^. Diagonal red dashed lines signal a sequence separation ≤ residue i, residue i + 34, equivalent to the longest-range NOE observed by NMR.

Fig. 6, *Left* shows the time-averaged “native” contacts observed in simulations of NCBD (bottom right) and the LEGO elements (top left). These maps reveal that H1H2 and H2H3 reproduce the native interactions present in full NCBD, albeit their contacts are slightly more transient. However, NCBD also engages in many nonnative interactions, including interactions that are longer range than the supersecondary structures recapitulated by LEGO elements (Fig. 6, *Right*). These “nonnative” interactions emerge as the differential factor in cooperatively biasing the conformational landscape of NCBD.

To estimate the energetic contributions from each set of interactions, we resorted to the helix–coil parameters from the LEGO analysis (Figs. 3–5) to calculate the statistical weight for forming a fully “native” α-helix conformation for each molecule. We then estimated the change in free energy from the ratio between the weight of a given combined LEGO element and the product of the weights of its building blocks (*SI Appendix*). We performed this calculation for the experimental and simulation data (Table 1). The experiments indicate that each set of pairwise tertiary interactions (helices 1 and 2, and 2 and 3) contributes ∼5 kJ/mol to 6 kJ/mol, which is comparable to the mean perturbation induced by single-point mutations on folded proteins (55). The interplay between helices 2 and 3 and tail contributes ∼3 kJ/mol more. The total NCBD stabilization amounts to ∼30 kJ/mol, which is comparable to the chemical denaturation free energies of two-state folding proteins, even though NCBD is an IPDP. However, such a comparison is misleading because the 30 kJ/mol for NCBD are referenced to a fully disordered ensemble (building blocks). In contrast, unfolded states have residual local structure (56). In general, the simulations produce much stronger pairwise tertiary interactions.

**Table 1.** Nonlocal energetic contributions

	H1−H2	H2−H3	H3−T	H2−H3T	NCBD	Coop.
Δ*G_exp_*	−5.4 ± 0.84	−6.0 ± 0.47	−0.9 ± 048	−8.6 ± 0.73	−30.7 ± 1.81	−16.8 ± 2.02
Δ*G_sim_*	−25.1	−10.2	−14.0	−29.7	−59.5	−4.6

The change in free energy (Δ*G*, in kilojoules per mol) of different contributions to the stabilization of the NCBD conformational ensemble as estimated from experiments and simulations (as explained in *SI Appendix*). Coop. indicates cooperativity. The errors shown for the experimental estimates correspond to 1 SD and have been obtained from the CIs from the helix–coil fits and their propagation to these composite parameters (as explained in *SI Appendix*).

To estimate the cooperative (nonadditive) contributions, we subtracted the pairwise interactions from the NCBD total stabilization. This calculation leads to an experimental estimate of ∼17 kJ/mol, and of ∼5 kJ/mol for the simulations (Table 1). The much smaller value for simulations is consistent with prior reports of MD simulations underestimating folding cooperativity (54, 57). As for the source of such cooperativity, it seems to arise from the simultaneous formation of tertiary interactions between helices 1 and 2, and 2 and 3, and nonnative interactions between helices 1 and 3 with the tail. The simulations also reveal that these sets of interactions compete with one another, resulting in alternating structural patterns. The conflict between sets of tertiary interactions, jointly with strong local propensities, explains why NCBD forms a highly dynamic ensemble rather than one 3D structure.

## Discussion

Since IDPs were first identified, we have faced the challenge of explaining how these proteins manage to integrate intrinsic disorder with the ability to select partners, fold upon binding, bind multiple partners, and switch among them in allosteric fashion. A key barrier has been the lack of methods that can dissect the conformational landscapes of IDPs in the absence of partners. Here we introduce a modular approach that is purposely designed to tackle this challenge (molecular LEGO) and apply it to the IPDP NCBD. The approach enables a direct comparison between experiments and simulations in a synergistic fashion. The molecular LEGO should, in principle, be easily generalizable to other IPDPs, and hence it adds a powerful tool for IDP research. In this regard, we outline some basic rules for its general application to disordered proteins.

1) A key element is the design of the LEGO elements. Ideally, one should use a structural ensemble of the unbound protein determined with one of the existing approaches for generating IDP ensembles from limited experimental restraints (58–60). As an alternative, one can use a structure of the IDP in complex with a partner, or even a secondary structure prediction profile (61).
2) Because these proteins are flexible/disordered, it is convenient to use a structure-promoting cosolvent as a thermodynamic variable, which also facilitates comparison with their folding upon binding behavior. TFE is a good option, particularly for IDPs that form α-helical structure (free or upon binding). Other alternatives are osmolytes, such as betaine and trimethylamine N-oxide (TMAO) (62), and salts, given that IDPs have very high net charges (13).
3) The conformational analysis should be carried out with techniques sensitive to the backbone conformation. Residue-averaged information is sufficient to address general mechanistic questions, as we do here with circular dichroism, or, alternatively, with infrared spectroscopy. NMR is an excellent choice, since it provides residue-specific structural information, but it could be too labor intensive to apply to all the LEGO elements and combinations.
4) It is essential to use a statistical thermodynamic treatment to analyze the experimental data, rather than assuming a two-state transition. Such treatment could be simple but should consider conformational entropy explicitly in terms of ensembles of microstates. Molecular simulations can test the physical significance of the choice of model used to analyze the experiments.

On a second front, the molecular LEGO study presented here sheds much-needed light on key mechanistic questions related to the conformational behavior of IDPs in general, and of NCBD in particular. Our results demonstrate that the amino acid sequence of NCBD contains strong local signals that almost singlehandedly define the secondary structural elements present in the ensemble. This observation supports the hypothesis that the conformational behavior of IPDPs is connected to the energetics of downhill folding (23). The combined LEGO elements demonstrate that the few tertiary contacts observed by NMR in NCBD produce energetic biases that help promote an overall helix bundle fold. However, these energetic contributions are relatively small (∼5 kJ/mol to 6 kJ/mol for each set of pairwise tertiary interactions: helices 1 and 2, and 2 and 3). From simulations, we find that the native tertiary contacts do form frequently but are transient (Fig. 6). These results explain the puzzling observation of specific long-range NOEs on an otherwise molten globule–like ensemble (29).

The behavior of full NCBD relative to the LEGO elements provides other important clues about IPDP energetics and folding landscapes. For instance, the tertiary interactions between helices 1 and 2, and 2 and 3, cooperate in the stabilization of NCBD's helix-bundle fold (mostly via the stabilization of helix 2). But we find that NCBD is much more ordered than expected from just its local and “native” pairwise tertiary interactions. Specifically, our experimental analysis reveals an extra ∼17 kJ/mol stabilization of the NCBD ensemble. That is, the structural factors used to calculate the NMR structure (local conformation and a few long-range NOEs) amount to less than 50% of the total ensemble energetics (Table 1). We find evidence of several such “nonnative” factors. The C-terminal tail, which is fully disordered in the NMR ensemble, turns out to be a major player. The tail alone elongates helix 3, but the interactions of helices 2 and 3 block such an extension and keep the tail disordered (H3T vs. H23T in Fig. 4). The tail can also interact with helix 1, resulting in end-to-end contacts (Fig. 6, *Right*) that stabilize helix 1 and form one helix turn on the tail. This helix turn is disconnected from, and bent relative to, helix 3. The end of helix 1 also interacts with the start of helix 3 in parallel fashion (Fig. 6, *Right*), which involves breaking many of the “native” interactions between helices 1 and 2, and 2 and 3. The pivotal role of the tail is highlighted by comparing our results with previous simulations of NCBD in which the tail was truncated (25). We note that all of these “nonnative” factors can be inferred from, or are consistent with, the LEGO experiments. They are, however, most evident in the simulations. This synergy highlights the importance of combining experiments and simulations in IDP research.

The picture that emerges from our dissection of the NCBD energy landscape is one of a protein with strong local structural biases and a tug of war between sets of tertiary interactions, each stabilizing a distinct conformational subensemble. Hence, the apparent disorder of NCBD arises from the conflict between competing tertiary interactions, which makes NCBD dynamically alternate between subensembles with slightly different fold architecture. This behavior is in stark contrast with the usual interpretation of disorder as indicative of absent tertiary interactions. Remarkably, the conformational properties we find in NCBD reveal an internal mechanism for driving its sophisticated, multipartner, folding upon binding behavior. The 3D structure of NCBD in complex with p53-TAD (38) is fully consistent with the “native” subensemble in which helices 1 and 3 interact with helix 2 but do not interact with each other, and the tail is disordered. These conformational biases are recapitulated by the LEGO elements H1H2, H2H3, and T. In contrast, ACTR and NCBD form an intertwined complex in which helices 2 and 3 of NCBD are set apart by ACTR, and helix 3 elongates onto the tail (8), precisely as we see in H3T and H23T. Finally, the “nonnative” interactions of helix 1 with helix 3 and tail are fully consistent with the structure that NCBD forms in complex with the stably folded IRF3 (42).

Summarizing, the NCBD folding landscape has built-in energetic biases that cooperate and compete to stabilize the various conformational subensembles that NCBD forms in complex with structurally diverse partners. This behavior uncovers an internal folding mechanism to select partners and modulate affinity that is likely essential for NCBD's recruiting role as transcription coactivator (12). The mechanism we report for NCBD is indicative of a conformational rheostat. It also demonstrates that the molecular LEGO approach can be used to map out subtle energetic biases on IPDPs, which are possibly essential to their biological function.

## Materials and Methods

An extended description of materials and methods is provided in *SI Appendix*.

### NCBD and Lego Elements.

Full NCBD was produced by recombinant means as a His-tag fusion and purified by affinity and reverse phase chromatography. Peptides corresponding to the eight Lego elements and combinations were chemically synthesized by Bio-Synthesis Inc.

### Experimental Conformational Analysis.

The conformational properties of NCBD and LEGO elements were characterized using far-UV circular dichroism spectra as a function of the helix promoting agent TFE. The spectra were analyzed using singular value decomposition to determine the average number of helical residues per condition. Each CD spectra vs. TFE dataset was analyzed with a tripartite helix/coil transition model in which the average number of helical residues at any given condition arises from the combination of three types of residues: PH, RC, and the elongation and nucleation of the TFE-sensitive IH. The effect of TFE was modeled to increase elongation in sequence independent manner as $s_{*}=s\left(1+1.75{\text{Φ}}_{\mathrm{TFE}}\right)$.

### Computational Conformational Analysis.

Molecular dynamics simulations in explicit solvent were performed using the GROMACS package, the Charmm22* force field, and the TIP3P water model. We obtained a total of 24 μs of simulation time for NCBD, 6 μs for H12 and H23T, and 4 μs for all the other peptides. All trajectories were analyzed to compute dihedral angles, hydrogen bonds, fraction of native contacts, time-averaged contact maps, and residue-specific helix elongation and nucleation parameters.

## Supplementary Material

Supplementary File

## Data Availability

All study data are included in the article and/or supporting information.
