# Supplementary material for: A modular approach to map out the conformational landscapes of unbound intrinsically disordered proteins
Source: Proc Natl Acad Sci U S A. 2022 Jun 3;119(23):e2113572119. doi: 10.1073/pnas.2113572119 (PMC9191344; doi:10.1073/pnas.2113572119)
Supplement: Supplementary File [file pnas.2113572119.sapp.pdf]

PNAS
www.pnas.org

Supplementary Information for

A Modular Approach to Map Out the Conformational Landscapes of Unbound Intrinsically Disordered Proteins

Thinh D.N. Luong[#,1,2], Suhani Nagpal[#,1,3], Mourad Sadqi[1,4] & Victor Muñoz[1,2,3,4,*]

Email: vmunoz3@ucmerced.edu

This PDF file includes:

Supplementary text
Figures S1 to S7
Table S1 to S2
SI References

Methods

Recombinant Protein Expression and Purification. The gene encoding for the full NCBD protein was cloned as a His-tag fusion in the bacterial expression vector pBAT4, which is designed to facilitate the expression of unstable and disordered proteins. The plasmid was transformed into *E. coli* BL21(DE3) competent cells. Transformed cells were grown in LB broth at 310 K until the optical density at 600 nm reached a value of 1.2-1.4, followed by induction with isopropyl-β-D-thio-galactopyranoside (IPTG) at 291 K overnight. The cells were then harvested by centrifugation at 8,000 rpm for 30 min. The pellet was resuspended until homogeneous in 20 mM phosphate buffer at pH 7.5. Cell lysis was carried out using the freeze-thaw method (6 cycles) followed by an ultracentrifugation step at 35,000 rpm for 30 min. After ultracentrifugation, the supernatant was collected and loaded onto a Nickel-column (His-Trap) as first step in its purification by affinity chromatography. The loaded column was washed with binding buffer (20 mM Tris 150 mM NaCl, 10 mM Imidazole pH 7.5) followed by elution with a gradient from 0% to 100% of elution buffer (20 mM Tris 150 mM NaCl, 500 mM Imidazole pH 7.5). Fractions containing the NCBD protein were pooled and subjected to a second round of HPLC purification on a reverse phase (RP) column using a 0%-95% acetonitrile gradient with 0.1% trifluoroacetic acid (TFA). All fractions containing pure NCBD were pooled, lyophilized, and stored at 253 K. Protein purity was assessed by SDS-PAGE and verified by electrospray mass spectrometry.

Peptide synthesis. Peptide molecules corresponding to the 4 building blocks and 4 combinations (sequences given in Figure 1) were chemically synthesized by Bio-Synthesis Inc. (Texas). Peptide purity was determined to be higher than 95% by RP-HPLC and mass spectrometry. Peptides were synthesized with acetylation and/or amidation at the N- and C-terminus, respectively, when the end of peptide corresponded to a non-terminal position in NCBD: H1 and H12 (free, amidated); H2, H3, and H23 (acetylated, amidated); T, H3T and H2H3T (acetylated, free).

Far-UV circular dichroism (CD) spectroscopy. Far UV-CD spectra were recorded from 190 nm to 250 nm with 1 nm resolution and 2 nm bandwidth at 278 K on a Chirascan CD spectrometer from Applied PhotoPhysics Ltd. (UK) equipped with a temperature controller system. A rectangular cuvette with 1 nm pathlength was used to hold the peptide/protein samples. NCBD samples were prepared at 30 μM, and all the peptides were prepared at 50 μM, except H2 which was prepared at 25 μM due to its lower solubility in 20 mM Tris-HCl. Actual samples were prepared by dilution from a 500 μM stock solution for peptides (300 μM for NCBD) previously prepared in the same buffer. TFE titrations were performed as follows: 200 μL of the initial sample was added to the cuvette, temperature equilibrated for 10 minutes, and the CD spectrum was acquired. Then the volume of TFE required to reach a 0.05 TFE (volume fraction) solution was added. After mixing, the sample was inserted into the cuvette holder, temperature equilibrated for 10 min, and then recording of the spectrum. The same process was repeated iteratively to reach a final TFE volume fraction of 0.5 in 0.05 increments. The protein concentration was corrected for each step to account for the volume changes resulting from TFE addition. The CD spectra were baseline subtracted (spectrum of a sample of the same buffer). All experiments were done in duplicate. The final protein concentrations of all the samples were calculated relative to an internal reference to minimize errors (due to the low or inexistent molar extinction coefficient at 280 nm of NCBD and peptides) using a three-step procedure: 1) the initial concentration of each sample was estimated from the concentration of the stock solution, which was determined either by absorbance at 280 nm for NCBD (two tyrosine residues, ε*(280 nm)* = 2x1,280 $M^{-1}cm^{-1}$) or by weight using an analytical scale for all of the peptides; 2) a correction factor based on the ratio of the absorbance at 195 nm (very strong signal: ε*(195 nm)* ≈ Nx~5,000 $M^{-1}cm^{-1}$, where N is the number of peptide bonds in the molecule) of each sample relative to that of H1 used as internal reference (corrected by the ratio of peptide bonds: N_x/N_{H1}) was applied to minimize pipetting errors; and 3) correction of the changes in total volume due to the TFE added to the sample.

Singular Value Decomposition (SVD). The set of experimental CD spectra as a function of TFE were organized as an M x N matrix, with M being the set of wavelength values and N the TFE volume fractions. Each element in the matrix represents the molar ellipticity value of the molecule at a given wavelength and TFE volume fraction. We used molar ellipticity instead of the commonly used mean residue ellipticity to obtain the absolute α-helical content of the molecule of interest,

which is unaffected by the number of non-helical peptide bonds that the molecule contains. The matrix of molar ellipticities was decomposed by SVD:

$$A = USV^T \qquad (1)$$

where U contains the spectral components, S is the diagonal matrix of singular values in decreasing ranking order, and V is a matrix with the amplitude of the U components as a function of TFE volume fraction. The first component corresponded to a pure α-helix CD spectrum (see Figure S7), and hence its amplitude (first column of V) represents the changes in helical content as function of TFE, which can be converted onto the average number of helical residues, $\langle k \rangle$, using the equation:

$$\langle k(\Phi_{TFE}) \rangle = V_1 \cdot U_1(222\ nm) \cdot S_1/(-39{,}500\ deg.cm^2.dmol^{-1}) \qquad (2)$$

where the denominator is the molar ellipticity of one helical peptide bond within a fully formed α-helix.

Helix-Coil treatment. We describe the formation of helical structure using the Zimm-Bragg helix-coil theory. In the Zimm-Bragg model, each peptide bond can be in either helical conformation (h) or coil (c), and helix formation occurs by a process of nucleation (cost of forming the first helical hydrogen bond, defined by the parameter σ) and elongation (defined by the parameter s). With this definition and using the coil as reference state, the statistical weight matrix is defined as:

$$\mathbf{M} = \begin{pmatrix} 1 & \sigma s \\ 1 & s \end{pmatrix} \qquad (3)$$

for which the partition function is

$$q = (1,0)\mathbf{M}^n \begin{pmatrix} 1 \\ 1 \end{pmatrix} \qquad (4)$$

where n is the number of peptide bonds in the molecule. The average number of helical residues is simply calculated as:

$$\langle k \rangle = n(s/\lambda_1)(1/2)\{1 + [(s-1) + 2\sigma]/[(1-s)^2 + 4\sigma s]^{1/2}\} \qquad (5)$$

where λ_1 is the largest eigenvalue of the statistical weight matrix:

$$\lambda_1 = \{(1+s) + [(1-s)^2 + 4\sigma s]^{1/2}\}/2 \qquad (6)$$

This treatment is for a homopolymer. To adapt it to heteropolymers and introduce the effect of TFE (TFE titration), we use the tripartite helix-coil model summarized in Figure 2, in which the heteropolymer sequence is divided into three types of units according to their average nucleation and elongation parameters: peptide bonds that are fully helical in water (PH), peptide bonds that are TFE-sensitive (i.e., inducible helix, IH) and peptide bonds that remain coil at all concentrations of TFE (RC). Using these definitions, we can calculate the average number of helical residues of a given peptide/protein as a function of TFE with the equation:

$$\langle k(\mathrm{TFE}) \rangle = \mathrm{PH} + \mathrm{IH}(s_*/\lambda_{1,*})(1/2)\{1 + [(s_*-1) + 2\sigma]/[(1-s_*)^2 + 4\sigma s_*]^{1/2}\} \qquad (7)$$

where $s_* = s(1 + 1.75\Phi_{TFE})$ and $\lambda_{1,*}$ is the largest eigenvalue of the statistical weight matrix at each TFE volume fraction. The effect of TFE is generic, that is, independent of the protein sequence. Equation 7 provides the basis for the fits to the experimental data presented in Figures 3-5.

Estimating pairwise tertiary interactions and cooperativity. In the Zimm-Bragg model, the statistical weight (*w)* of a given helical conformation is given by $w = \sigma s^i$, where *i* is the number of helical peptide bonds. We can calculate the statistical weight expected for a fully folded molecule containing two helical elements (molecular LEGO's building blocks) that are not interacting with one another as the product of the statistical weights of the fully formed helical elements. Hence, the contributions from tertiary interactions between the two elements can be obtained from the ratio between the statistical weights of the entire molecule divided by the product of the weights of its separated elements as:

$$\Delta G_{mn} = -RT\ln\left(\frac{w_{mn}}{w_m w_n}\right) \qquad (8)$$

where $w_m = \sigma_m s_m^{k_m}$ and $w_n = \sigma_n s_n^{k_n}$ are the statistical weights of the fully-induced helical conformation of building blocks *m* and *n*, and $w_{mn} = \sigma_{mn}{}^2 s_{mn}^{k_{mn}}$ is the statistical weight of a molecule containing building blocks *m* and *n* in full helical conformation. In these expressions, *k* is the number of residues that need to become helical to form the helix(es) defined in the NCBD NMR ensemble

(dashed lines in Figures 3-5), that is, $k = H_{NMR} - \text{PH}$. We used this procedure to calculate pairwise interactions between helices 1 and 2 and helices 2 and 3. For the tail (T), we considered that its effect on a combined molecule is to extend helix 3 rather than nucleating a new one (w_{3T} only includes 1 nucleation site and w_{23T} includes 2). After the pairwise tertiary interactions have been estimated, the same calculation can be carried out for the entire protein to estimate the overall folding cooperativity. In this case, the fully formed conformation includes three helices, and hence $w_{NCBD} = \sigma_{NCBD}{}^{3} s_{NCBD}^{k_{NCBD}}$, relative to the product of the statistical weights of the four elements. The overall folding cooperativity is finally obtained as:

$$\Delta G_{coop} = \Delta G_{NCBD} - (\Delta G_{H12} + \Delta G_{H23T}) \qquad (9)$$

We performed these calculations for the experimental data using the helix-coil parameters given in the insets of Figures 3-5, and for the MD simulations using the nucleation and elongation parameters from Table S2.

Data Fitting and Error Analysis. The experimental data (average of two independent experiments) for each of the nine molecules were fitted to the tripartite helix-coil model (equation 7) using the non-linear least squares *lsqcurvefit* MATLAB function implemented with the trust-region-reflective algorithm. The estimated errors (one standard deviation) of the four fitted parameters for each molecule were obtained directly from the Jacobian matrix and the residuals of the best fit using standard error propagation analysis. For NCBD, we also carried out fits with the maximum helical length fixed to 46 residues, as means to decouple the determination of PH and IH in the analysis of this broad transition. This maximum helix length for NCBD was obtained from the sum of the experimental helical lengths (IH+PH) of H1, H2 and H3T (insets of Figures 3-4), which is also in very good agreement with the value obtained from the free fit (Figure 5). We also estimated the tripartite helix-coil model parameters and statistical errors from the mean and standard deviation of the parameters obtained from best fits to each individual experimental dataset of a given molecule. The mean and standard deviation of the parameters estimated from individual fits for the 9 molecules are given in Table S1.

We determined the statistical errors of the pairwise tertiary interactions and cooperativity values (Table 1) from the propagation of the errors of all the basic parameters involved in each calculation (σ, *s*, IH, PH, for all molecules involved in each calculation with equations 8 and 9). Particularly, we performed numerical simulations in which a distribution of values for each basic parameter was produced (generating a collection of 500 random samples) according to their statistical errors (insets in Figures 3 to 5). These distributions were combined to generate 500 samples for each derived parameter (pairwise interaction or cooperativity) using equations 8 and/or 9. The propagated statistical errors (one standard deviation) are given in Table 1 together with the best estimate (mean).

To test the statistical significance of the tripartite helix-coil model, we performed a F-test regression analysis relative to the performance in fitting the data of a standard, statistically simpler, homopolymer helix-coil model with only 3 parameters: σ, *s*, and maximal helix length (which does not need to be the entire length of the molecule to allow for the presence of non-helical flanking residues). The F-statistic is then computed from the squared residuals obtained from the best fits performed with both models as:

$$F = \frac{(SS_1 - SS_2)/(df_1 - df_2)}{SS_2/df_2} \qquad (10)$$

where SS_1 and SS_2 are the sum of squared residuals for the simpler (fewer parameters) and the more complex (more parameters) models, respectively; and df_1 and df_2 are the respective number of degrees of freedom (number of datapoints minus number of parameters). From this analysis on the entire NCBD molecular Lego dataset, we obtained a probability p=0.0059 that the simpler model could reproduce the experimental data at a level comparable to that of the complex model; thus, confirming the statistical significance of the tripartite helix-coil model (and of the presence of short regions with intrinsically high helical content in the NCBD sequence) in describing the helix-coil behavior of the NCBD-derived fragments and entire protein with better than 99% confidence.

All-atom MD simulations. We carried MD simulations in explicit solvent using the GROMACS package(1-3), and the Charmm22* force field (4). Water molecules were described using the TIP3P

model. Periodic boundary conditions were used, and long-range electrostatic interactions were treated with the Particle Mesh Ewald (PME) (5) summation using a grid spacing of 0.16 nm combined with a fourth-order cubic interpolation to derive the potential and forces in-between grid points. The real space cutoff distance was set to 1.2 nm, and the van der Waals cutoff to 1.2 nm. The bond lengths were fixed(6), and a time step of 2 fs was used for the numerical integration of the equations of motion. Coordinates were recorded every 10 ps.

For NCBD, we performed two separate 12 μs trajectories starting from the lowest energy structure of the NCBD NMR ensemble (PDB ID: 2KKJ). The protein was placed in a dodecahedral water box (volume = 262.38 nm^3) large enough to contain the protein and at least a 1.0 nm layer of solvent on all sides. The structure was solvated with 8,216 water molecules, and six Cl^- ions were added to neutralize the system. The starting coordinates for the 8 NCBD fragments (as defined in Figure 1) were extricated from the protein's PDB file. The fragments were acetylated and/or amidated as needed to replicate the chemically synthesized peptides (H1, H12 free and amidated; H2, H3, H23 acetylated and amidated; T, H3T, H23T acetylated and free). The CHARMM22* force field was then adjusted to include the parameters for N-acetylation and C-amidation. Box dimensions were kept sufficiently large to account for the high flexibility and large-scale motions expected on these peptides. Two 2 μs trajectories were performed for each fragment (three 2 μs trajectories for the larger fragments H12 and H23T).

In all cases, the starting structure was subjected to energy minimization using the steepest descent method. All systems were equilibrated at a constant temperature of 310 K utilizing the two-step ensemble procedure (NVT and NPT). First, the system was subjected to NVT (constant number of particles, volume, and temperature) equilibration for 100 ps with the position of the protein restrained, followed by NPT (constant number of particles, pressure, and temperature) equilibration for 2 ns each. The simulations were subjected to the modified Berendsen thermostat with a 0.1 ps relaxation time(7) to maintain the temperature. The structures were then subjected to Parrinello-Rahman with 0.2 ps relaxation time for pressure coupling(8) at 1 bar before the production run was started. All the simulations were run on the Triton Shared Computing Cluster (TSCC) at the San Diego Supercomputing center (SDSC).

Analysis of MD simulations. The number of native contacts per residue was calculated from each MD trajectory with a 1 ns time step and using the NMR structure as the reference of native contacts. Contacts were defined using a 0.5 nm cutoff between any two pairs of heavy atoms that are at least 3 residues apart in the sequence. The number of native contacts trajectory was then converted into the fraction of native contacts (*Q*). We used the peptide bonds as basic conformational unit to compare with experimental data analyzed with the Zimm-Bragg model. Each trajectory was then analyzed to assign each peptide bond of the simulated molecule to either helix or coil state at each time frame. The helical state (*H*) was defined according to the local conformation (dihedral angles) and backbone hydrogen bonding status. These processed trajectories were finally used to calculate the number of helical residues per time frame, and the average fraction helix per residue for each molecule.

Analysis of dihedral angles. We classified the conformation of a peptide bond unit based on its flanking ψ and ϕ angles. Particularly, we defined a helical peptide bond (*h*) when its dihedral angles are $-50° < \psi < -17°$ and $-80° < \phi < -50°$, and coil peptide bond (*c*) as everything else.

Analysis of hydrogen bonds. A hydrogen bond between residues *i and i*+4 was considered formed when the donor-acceptor distance was < 0.35 nm and the donor-hydrogen-acceptor angle >160°. We computed every hydrogen bond formed at each time frame using the MD Analysis python toolkit: we first evaluated all possible hydrogen bonds per time frame, and then every time a *i, i*+4 hydrogen bond was formed according to our criteria, we assigned a hydrogen-bonded state (h_{HB}) to peptide bonds *i*+1, *i*+2, and *i*+3.

Computing helix nucleation and elongation. We define the elongation parameter (*s*) as the equilibrium constant between the helix and coil states of the central peptide bond in a triplet. For a given time frame, the helix state of the central peptide bond is any of the following: [c $\underline{h}$ h], [h $\underline{h}$ h], [h $\underline{h}$ c] or [c $\underline{h}$ c]; and the coil state is either [h $\underline{c}$ c], [h $\underline{c}$ h], [c $\underline{c}$ h] or [c $\underline{c}$ c]. For a predefined helical segment, s is simply the average of the elongation for all the peptide bonds within it.

The nucleation parameter (σ) is defined as the equilibrium constant for the formation of the first helical (*i, i*+4) hydrogen bond (flanked by coil peptide bonds). To calculate σ, we used a rolling window of 7 peptide bonds and defined nucleation on the third peptide bond ($\tilde{h}$) as:

$$\sigma = \frac{1}{t}\sum(cc\tilde{h}hhcc) \qquad (11)$$

where *t* is the number of time frames in the trajectory. The final parameters for one molecule were determined as the average over all the available MD trajectories.

Time-averaged contact maps. To calculate the time-averaged contact maps we considered that a contact is formed at any given 1 ns interval when at least one atom of residue i is within a cutoff distance of 0.5 nm of at least one atom of residue j (where j ≥ i+3). Native contacts were defined on the basis of the atomic coordinates of the NMR structure.

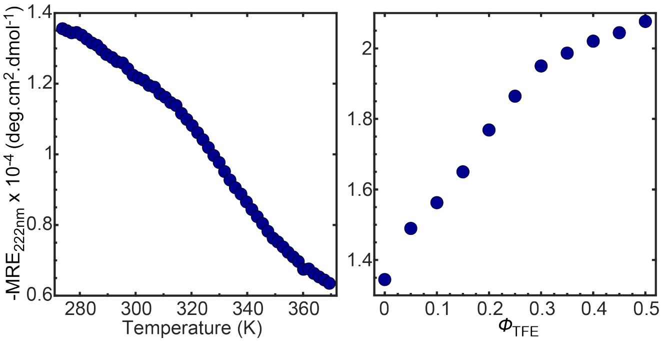

Fig. S1. Folding thermodynamics of NCBD. Left) thermal denaturation experiment of NCBD monitored by circular dichroism (CD) at 222 nm. Right) equilibrium stabilization of NCBD induced by 2,2,2-trifluoroethanol (TFE) and monitored from the CD signal at 222 nm at 278 K.

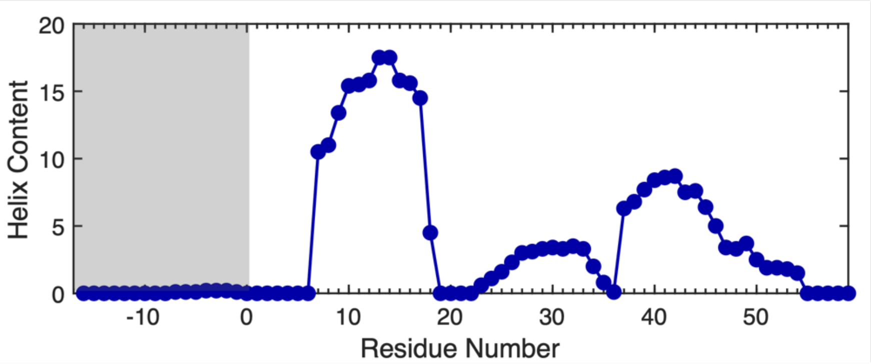

Fig. S2. Predicted NCBD helical content from AGADIR of the NCBD construct including the His tag (framed in a gray swath). The prediction shows that there is no helical propensity in the N-terminal region until it reaches Pro7 within the NCBD sequence (as shown in Figure 1). AGADIR also shows

that the NCBD sequence has clear propensity to form three α-helices located in the same precise regions that are helical as determined by NMR experiments (Kjaergaard M. et al., 2010).

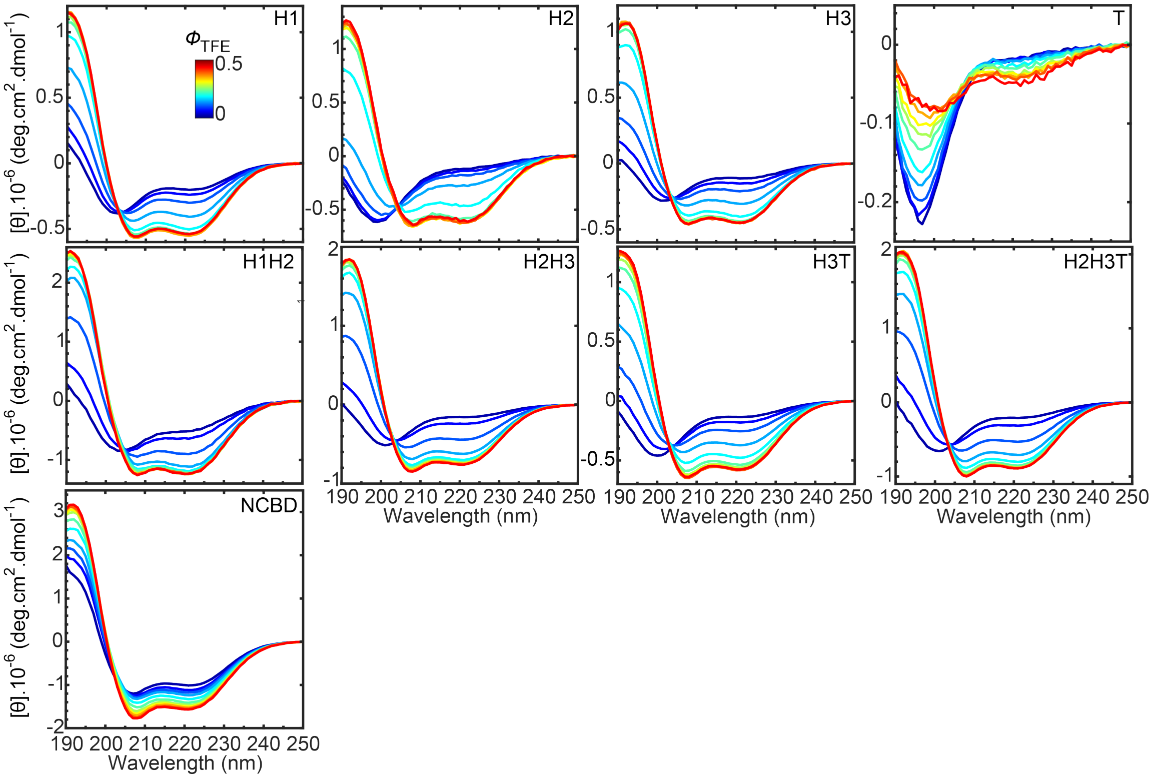

Fig. S3. Far UV-CD spectra (in molar ellipticity units) of all molecular LEGO elements and full NCBD as a function of TFE volume fraction (ϕ_{TFE} from 0 to 0.5) at 278 K.

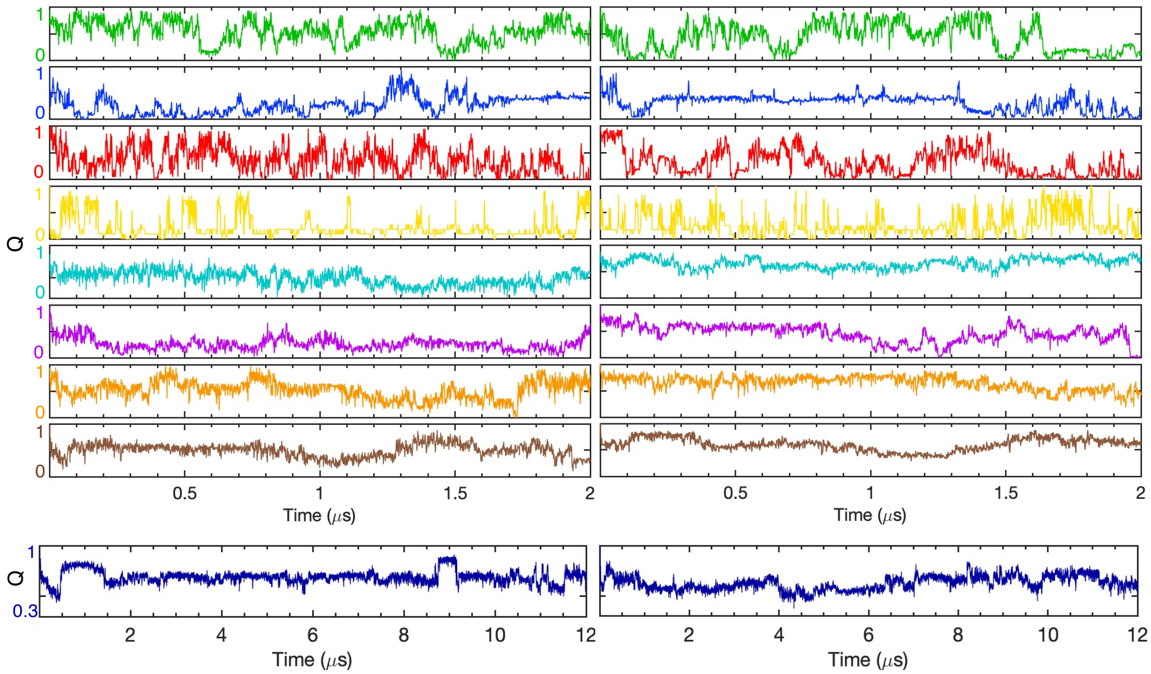

Fig. S4. Time evolution of the fraction of native contacts (Q) sampled in representative MD trajectories of all 8 fragments (top panel) and full-length protein (bottom panel).

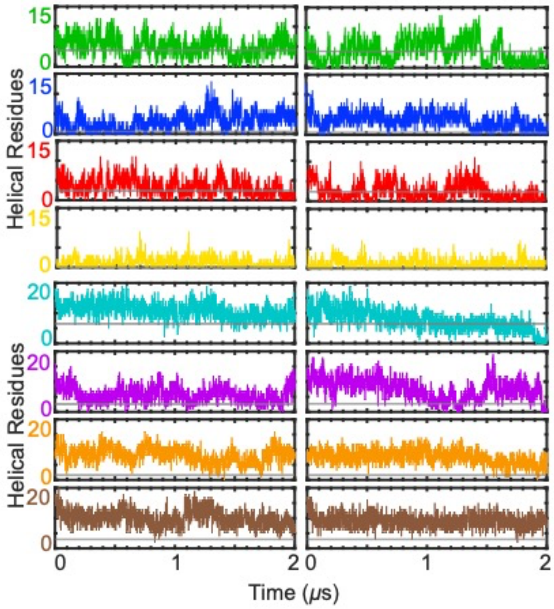

Fig. S5. Number of helical residues as a function of time for two sets of MD trajectories of all building blocks (top) and their super-secondary combinations (bottom). The first set is also depicted in Figure 3 and 4 respectively. Color code as in Figure 1.

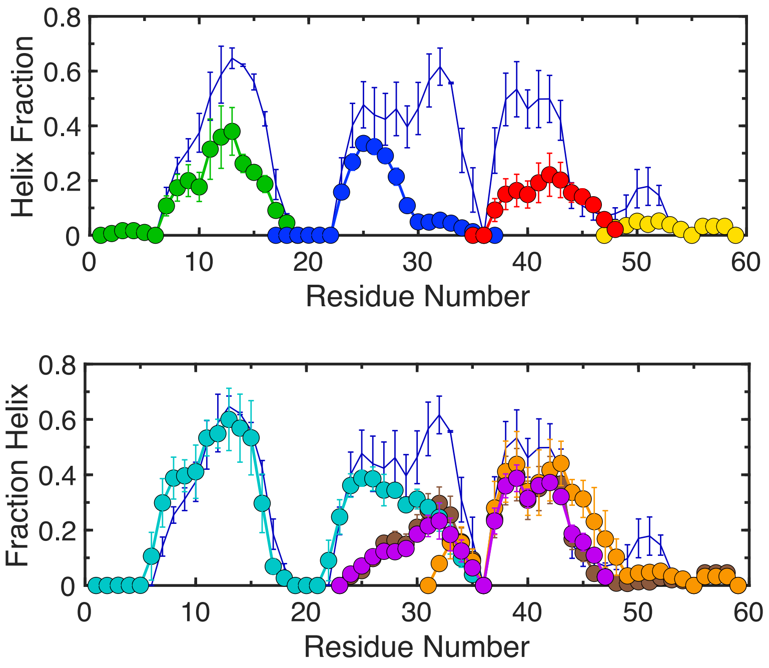

Fig. S6. Helix fraction per residue based on hydrogen bond definition for all MD ensembles. Top: building blocks. Bottom: combinations of building blocks. The full-length protein is shown with thin, navy blue lines as reference. Color code as in Figure 1.

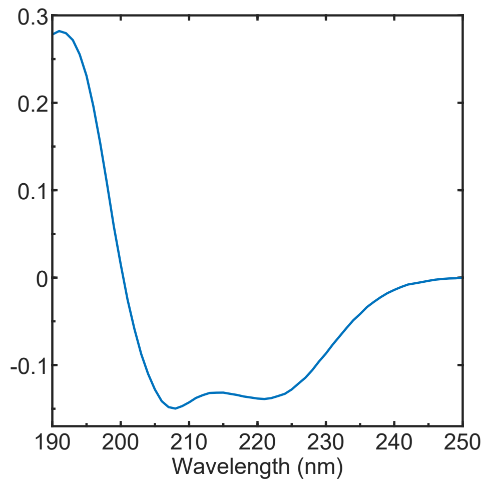

Fig. S7. First spectral component (U) from the singular value decomposition of the far-UV circular dichroism spectra of the entire experimental dataset (4 LEGO building blocks, 4 LEGO combinations and full NCBD as a function of TFE volume fraction).

Table S1. Helix-coil parameters and confidence intervals calculated from the mean and standard deviation of the best fit parameters from independent fits to individual datasets.

	H1	H2	H3	T	H1H2	H2H3	H3T	H2H3T	NCBD
<σ> (10^3)	3.5	1.4	3.5	7.1	1.9	2.9	4.6	4.8	36.5
σ error (10^3)	± 0.24	± 0.05	± 0.20	± 0.37	± 0.51	± 0.04	± 0.09	± 0.4	± 0.84
<*s*>	0.81	0.77	0.80	0.68	0.87	0.85	0.80	0.85	0.87
s error	± 0.004	± 0.002	± 0.005	± 0.007	± 0.005	± 0.005	± 0.004	± 0.004	± 0.046
<IH>	12	15.5	11.2	1.1	25.6	22.3	16.3	26.1	32.5
IH error	± 0.51	± 0.35	± 0.43	± 0.04	± 0.31	± 0.06	± 0.1	± 0.16	± 5.2
<PH>	3.4	0.3	1.8	0.0	4.6	0.67	0.5	0.0	13.4
PH error	± 0.01	± 0.05	± 0.12	± 0.0	± 1.07	± 0.09	± 0.04	± 0.0	± 3.8

Table S2. Helix–coil model parameters calculated from MD simulations of all components and NCBD (details in Methods).

s	0.63	0.39	0.39	0.14	0.87	0.5	0.8	0.8	1.2
σ	0.018	0.019	0.021	0.004	0.025	0.023	0.018	0.018	0.024

SI References

1. M. J. Abraham *et al.*, GROMACS: High performance molecular simulations through multi-level parallelism from laptops to supercomputers. *SoftwareX* **1-2**, 19-25 (2015).
2. H. J. C. Berendsen, D. van der Spoel, R. van Drunen, GROMACS: A message-passing parallel molecular dynamics implementation. *Comput. Phys. Commun.* **91**, 43-56 (1995).
3. S. Páll, M. J. Abraham, C. Kutzner, B. Hess, E. Lindahl, Tackling Exascale Software Challenges in Molecular Dynamics Simulations with GROMACS. in *Solving Software Challenges for Exascale*, eds S. Markidis, E. Laure (Springer International Publishing, Cham), 3-27 (2015).
4. S. Piana, K. Lindorff-Larsen, D. E. Shaw, How robust are protein folding simulations with respect to force field parameterization? *Biophys. J.* **100**, L47-L49 (2011).
5. T. Darden, D. York, L. Pedersen, Particle mesh Ewald: An N·log(N) method for Ewald sums in large systems. *J. Chem. Phys.* **98**, 10089-10092 (1993).
6. B. Hess, H. Bekker, H. J. C. Berendsen, J. G. E. M. Fraaije, LINCS: A linear constraint solver for molecular simulations. *J. Comput. Chem.* **18**, 1463-1472 (1997).
7. H. J. C. Berendsen, J. P. M. Postma, W. F. van Gunsteren, A. DiNola, J. R. Haak, Molecular dynamics with coupling to an external bath. *J. Chem. Phys.* **81**, 3684-3690 (1984).
8. M. Parrinello, A. Rahman, Crystal Structure and Pair Potentials: A Molecular-Dynamics Study. *Phys. Rev. Lett.* **45**, 1196-1199 (1980).
